# Retraction: GATA4 inhibits cell differentiation and proliferation in pancreatic cancer

**DOI:** 10.1371/journal.pone.0348036

**Published:** 2026-04-27

**Authors:** The PLOS One Editors

After publication of this article [1], concerns were raised regarding Figs 2 and 3. Specifically:

- The Fig 2A GATA4 panel appears similar to lanes 3 and 4 in the Fig 3A GATA4 panel when rotated 180° despite these panels representing different experimental conditions.
- The Fig 2A GAPDH panel appears similar to lanes 1 and 2 in the Fig 3A GAPDH panel despite these panels representing different experimental conditions.
- Lanes 2 and 4 in the Fig 3A GATA4 panel appear similar to each other.

First author YG stated that these issues stemmed from improper data organisation workflows, and that consequently, several blot bands were inadvertently misused or repeated during figure assembly, but was not able to provide the original underlying images for Fig 2A. During post-publication discussions, original western blot images were provided for Fig 3A; however, these do not appear to match the corresponding panels in [1].

In light of the above concerns, which call into question the integrity and reliability of the results in [1], the *PLOS One* Editors retract this article.

YG did not agree with the retraction. LZ, AZ, XC, PG, and QZ either did not respond directly or could not be reached.

## References

[1] Gong Y, Zhang L, Zhang A, Chen X, Gao P, Zeng Q. RETRACTED: GATA4 inhibits cell differentiation and proliferation in pancreatic cancer. PLoS One. 2018;13(8):e0202449. doi: 10.1371/journal.pone.0202449 30142155 PMC6108473

